# Spending, Mending, or Ending

**Published:** 1890-03-22

**Authors:** 


### The Hospital

A Weekly Journal of

Science, Medicine, Nursing, and Philanthropy.

*For Hospitals, Asylums, Medical Practitioners, Students, Nurses, Families, Parishes, Congregations, and the Charitable Public.*

Vol. VII.—No. 182.] [March 22, 1890.

### Spending, Mending, or Ending.

One of the easiest things in the world is to spend money; one of the most difficult is to spend it well. Nothing could be more trite than these observations, and nothing could be more true. What an enviable housewife is she who knows how to make a pound purchase thirty shillings' worth of household necessaries. The art of spending requires genius for its perfection; the perfect spender, like the poet, is born and not made. Almost everybody rails at extravagance, and almost everybody is extravagant. Many a husband is overwhelmed with astonishment at the way in which his wife makes the money vanish, and at the poor results she produces for his inspection and comfort. Many a wife, on the other hand, wonders what her husband does with his money, for, as she complains, he gives her very little, and yet he never seems to have a penny in his pocket. We are called upon once more in the course of our duty to consider the question of Hospital expenditure, and the problem seems so difficult, not to say hopeless, of solution, that we have felt it necessary to fortify ourselves at the outset with these general reflections on the fundamental characteristics of human nature.

The Hospitals Association conceives that one of its duties is to go about, like Diogenes, with a lantern in its hand, to see if it can find a perfectly administered Hospital. The deputy-chairman of that Association has a special zeal for administrative perfection; and, with slight intermissions from January to December, he seeks to make such perfection universal. He recognises, as everybody does, that money is the key to the situation. In Hospital work, as in so many other of life's circumstances, the Alpha and the Omega is money. The reading of papers is one of the diversions of the Association, as it is of many learned and portentous societies; and the reading of papers may be a very pleasant thing if the papers do not happen to be too critical, and if one is not oneself the unfortunate subject of them. At the February meeting of the Association a paper was read by the deputy-chairman, which purported to discuss "The Hospital Problem." But it very soon appeared that the particular Hospital Problem then to be considered was simply our old friend "Money" under a new name. To consider in any detail the many points brought out in the paper would be impossible. We shall, therefore, select for some remarks one important aspect of the case—the aspect of comparative expenditure.

Somebody has said that because the sun shines every day, therefore, to a common-place mind the shining of the sun ceases to be wonderful. Whether this be so or not, it is certainly true that the most extraordinary contrasts and diversities in Hospital expenditure have long since lost their power of exciting even a languid interest in the average Hospital supporter. All that he craves is to be let alone. As an example of his hopeless indifference may be mentioned the fact that not a single person outside the Hospitals Association seems to have taken the least notice of the startling contrasts in the cost of Hospital maintenance revealed by Mr. Burdett. His paper separates British Hospitals into four groups—The Hospitals of London, of the English provinces, of Scotland, and of Ireland. Regarding for the moment these four groups as one, we find that some Hospitals spend more than £100 on each occupied bed per annum. It will scarcely be credited that at the opposite end of the scale there are several that spend only a little over £20 per bed per annum. In other words, there are Hospitals in which each occupied bed costs more than four times as much as in certain other Hospitals. If any one denies that this is a very peculiar circumstance, he is to be pitied. If any one says that it does not really matter whether or not a patient at one Hospital costs four times as much as a similar patient at a similar Hospital, he is probably one of those to whom nothing matters. If it does not matter to the ordinary Hospital subscriber, then the ordinary subscriber must be a very peculiar person indeed.

The Londoner may say that no comparisons can fairly be instituted between a large London Hospital and the Cottage Hospital of a small market town in a remote part of Scotland or Ireland. Let that be admitted. It cannot, however, be denied that it is of some concern to the Londoner if he finds the cost per bed per annum at one London Hospital quite double the cost per bed per annum at another London Hospital exactly similar in every respect. And this is what Mr. Burdett's paper has brought to light. Fifty-six of the Metropolitan Hospitals are divided into six groups. Of these fifty-six twenty-one spend more than £100 per bed per annum, three spend more than £90 per bed, six more than £80, eleven more than £70, nine more than £60, and six more than £50. The public ought to be curious to know what class of Hospitals the twenty-one that spend more than £100 per bed per annum each belong to. Are they General, or are they Special Hospitals? Are they large, or are they small? Nineteen of the twenty-one are special Hospitals, and they are for the most part very small.

Now if we were actually reforming the Hospital system instead of merely talking about it, we should stop here and ask two or three pertinent questions. First of all, why are there so many Special Hospitals in London? Secondly, why are they so costly? Thirdly, are Special Hospitals in such numbers indispensable to the well-being of the poor? Or, cannot all the necessities of the poor be met in some more economical way? These questions are entirely practical; and those who know anything about the matter never doubt that the answers to them are only of one kind. Whatever may have been the case in 1840, it is quite certain that in 1890 very few Special Hospitals are really needed. Most, if not all, the General Hospitals in London have special departments, and in consequence of their superior prestige those hospitals almost invariably command the best services of the best specialists in the medical profession. In addition to being superfluous, it can hardly be doubted that Special Hospitals as a class are also extravagant. For we find that although four of them can do their work for a little over £50 per occupied bed per annum each, nineteen others spend more than £100 each per occupied bed per annum. If, therefore, the question of the necessity for Special Hospitals is not now ripe for consideration, it seems doubtful if it will ever be ripe.

Provincial Hospitals are, on the whole, much less costly institutions than those of London. That will surprise nobody. Food is cheaper in the country, and so are the services of workers. Still the difference of expenditure between London and the country is greater than there seems to be any necessity for; and this difference is much more marked when the Special Hospitals of the country are compared with those of the metropolis. Whilst nineteen of the London Special Hospitals are placed in the group that spend more than a £100 per bed per annum, the most costly of the Provincial Special Hospitals belong to the group that spend something over £60 per bed per annum. Why this should be so it is impossible to imagine. But if the London Special Hospitals have anything to urge in extenuation, now is the time to urge it.

Scotland, as many people will be prepared to expect, manages her Hospitals much more economically than any other part of the United Kingdom. Of seventeen Scotch Hospitals, which have furnished particulars, not one spends more than £70 per occupied bed per annum. There are only four that spend more than £50. Thirteen of the seventeen fall into the group which spend something over £40 per bed. Ireland has one or two decidedly extravagant Hospitals, though for the most part she, too, shows a commendable capacity for economy.

We have given but two or three leading points of a paper that is full of the most interesting and important facts. It is plain, however, that certain classes of Hospitals, and particularly in London, spend extravagantly. What is to be done with them? They must be "mended or ended." The charitable institutions of this country have a noble history and a fair fame, of which the present generation of Hospital managers are the responsible heirs and custodians. Moreover, all Hospitals and all managers constitute a separate and distinct class, and are related to each other as a vast confraternity. The spendthrifts have no right to say to the economists "You shall not criticise us;" nor have the economists any right to say to the spendthrifts, "We do not care how black you are so long as our own hands are clean." Circumstances have brought together all classes of Hospitals and managers into one body, and as one body they appear to the world; as one body they are approved or condemned; and as one body they stand or fall. The spendthrifts, and those whose institutions are of doubtful or expired utility must look to themselves if they would not be mended or ended from the outside. But whatever action is taken by them, or whether they elect to fold their hands and sit still, the economists at any rate have but one choice: they must search for the best methods of administration and practise them; and they must never cease to proclaim from the house-tops that it is the duty of every man, both great and small, who is entrusted with the expenditure of public money, to do likewise.

